# Supplementary material for: A study of the impact of de-capacity policies on industry capacity utilization paths: Evidence from the Chinese steel industry
Source: PLoS One. 2023 Dec 15;18(12):e0295613. doi: 10.1371/journal.pone.0295613 (PMC10723696; doi:10.1371/journal.pone.0295613)
Supplement: S1 Data — (DOCX) [file pone.0295613.s001.docx]

Data for the explanatory variables, dependent variables, and control variables used in this paper can be accessed at the following link: https://figshare.com/s/bf3aae2165b6d002addf
