# Supplementary material for: A study of the impact of de-capacity policies on industry capacity utilization paths: Evidence from the Chinese steel industry
Source: PLoS One. 2023 Dec 15;18(12):e0295613. doi: 10.1371/journal.pone.0295613 (PMC10723696; doi:10.1371/journal.pone.0295613)
Supplement: Attachment — Submitted filename: Required Revisions.docx [file pone.0295613.s002.docx]

Dear Authors,

Hope this letter finds you well. Please find attached the required revisions that would improve your research paper before publication.

Regards

The reviewer

**Positive Aspects:**

- The article is well organized and provides valuable insights about research topic in the field. Moreover, the structure of the analysis is clear and logical.

- Research design is clear, comprehensive, and detailed. Also, the selection of variables is well justified and the integration between them and other factors enriches the analysis by explicitly examining the policy's effects on capacity utilization.

- Conclusions and implications provide a clear summary of the study's findings and offer actionable recommendations for policymakers.

**Suggestions for Improvements:**

**- Literature Review Section:**

- Adding more examples can enhance the research. E.g., the researchers can incorporate some practical examples or case studies from China and/or other real-world examples to improve the research practicality and relevance. These of course should be recent examples and cases.
- **Research Design:**
- Clarifying methodologies. For example, the use of a ***data envelope model*** is mentioned for measuring the capacity utilization rate. Therefore, providing more explanations and references regarding this model and how it's applied would enhance the clarity of the research design.
- Expanding on Data Sources: For example, providing more information on the reliability, representativeness, and accuracy of the data to enhance the research design.
- Discussing Limitations: For example, addressing potential limitations of the chosen methodologies, data sources, and the overall research design should add transparency and ensure a vigorous interpretation of results.
- **Conclusions:**
- Incorporating Quantitative Results: Incorporating specific quantitative results or statistics from the analyzed tests can enhance the credibility and clarity of the conclusions regarding the vitality of the chosen policy time breakpoint.
- Providing Citations for Theoretical Analysis: Providing references or citations to support the theoretical assertions can strengthen the argument and enhance academic and research rigor.
- Elaborating more on Industry Cultivation Mechanism and multi-dimensional collaborative supervision: One of the research recommendations is that the need for improving the industry cultivation mechanism and multi-dimensional collaborative supervision. Expanding on what this entails and how it can positively impact capacity utilization would provide more clarity.

- **Limitations:**
- Addressing Potential Limitations: Such as assumptions or constraints, to ensure a balanced view and promote transparency in the research.
- **Grammar and Style:**
- There are minor grammatical errors. Stylistic improvements can be made to enhance the overall flow and readability of the text.
