# Supplementary material for: A study of the impact of de-capacity policies on industry capacity utilization paths: Evidence from the Chinese steel industry
Source: PLoS One. 2023 Dec 15;18(12):e0295613. doi: 10.1371/journal.pone.0295613 (PMC10723696; doi:10.1371/journal.pone.0295613)
Supplement: Attachment — Submitted filename: Response to Reviewers.docx [file pone.0295613.s003.docx]

Dear Dr. Sweis and dear reviewers,

We would like to thank you for your efforts in reviewing our manuscript titled “A study of the impact of de-capacity policies on industry capacity utilization paths: Evidence from the Chinese steel industry”, and providing many helpful comments and suggestions, which will be all proved invaluable in the revision and improvement of our paper, as well as in guiding our research in the future. We have studied comments carefully and have made correction, and we hope that it will be met with approval (the replies are highlighted in blue). All revised portions are marked in yellow in the revised manuscript which we would like to submit for your kind consideration. Additionally, clean revised manuscript and supporting information are also uploaded.

The main corrections in the paper and the response to the comments are as following:

**Responds to the Academic Editor:**

- Please restructure the abstract and make sure to present the findings in a clear fashion.

**Response:** We would like to express our gratitude for this valuable suggestion. We promptly restructured the abstract to ensure that the findings are presented in a clear and concise manner, addressing this concern effectively. Thank you for your constructive feedback.

- Emphasize your contributions by comparing your work to other published work in the field.

**Response:** Thank you for your valuable suggestions. According to your suggestion, in the introduction, we have pointed out the shortcomings of existing literature and proposed the contribution of this paper to the shortcomings of existing research. With your help, the integrity of the paper has been further improved.

- Add a limitations section.

**Response:** We greatly appreciate your valuable suggestion. In the conclusion part, we add the limitations of the paper and the potential approaches for future research. And we hope this will enhance the clarity of our findings.

- The LR needs to be enhanced further by exploring international studies to clarify deficiencies in the current body of literature.

**Response:** Thank you for your valuable suggestions. According to your suggestion, we expand the literature review by including international research to help shed light on overcapacity issues that are of concern to countries around the world. This enhancement offers a more comprehensive perspective on our research topic, contributing to a deeper understanding of the subject matter.

**Responds to the reviewers’ comments:**

**Reviewer #1:** The abstract should present the study's findings in different wording, and personal pronouns should be avoided.

**Response:** Thank the reviewers for their valuable suggestions. As suggested, we have made changes to the article, while also checking the full text to avoid the use of personal pronouns.

The study effectively elucidates the background problem, but it falls short in clarifying the deficiencies present in the current body of literature. Consequently, it is advisable for the authors to emphasize the likely knowledge gaps and integrate these into their study results.

**Response:** It is our great honor to have your recognition of this work. Thank you for your question. According to your suggestion, in Section 1 (Introduction), we have pointed out the shortcomings of existing literature and proposed the contribution of this paper to the shortcomings of existing research. With your suggestions and help, the integrity of this paper has been improved.

The analytical framework and data envelope model should be made clearer to reflect the subject of the study and have in-depth bases from the literature.

**Response:** Thank you for your valuable comments. In response to your comments, we have thoroughly revised the analytical framework and data envelope model in our paper to improve clarity and ensure they align more effectively with the subject of our study. Additionally, we have incorporated more in-depth support from the existing literature to further strengthen these components. Your insights have been immensely valuable in enhancing the quality of our research. Thank you for your guidance and support.

A more comprehensive explanation of Figure 1 is needed, encompassing all the used components and their interconnections.

**Response:** We sincerely appreciate your suggestion. In order to facilitate readers' better understanding, we have further described Figure 1 in more detail, hoping that this can enhance the readability of the article and facilitate readers' better understanding.

The examination of Paths should be guided by the knowledge present in the existing literature.

**Response:** Thank you for your valuable suggestion. In parts 2.1 (Analytical framework) and 2.2 (Path analysis), we have provided corresponding references and relevant materials for the five proposed paths, hoping to strengthen the academic character of the paper.

The title "Explanatory Variables" was duplicated in both sections 3.1.1 and 3.1.2. To address this, the authors can provide an introduction of the components of the Explanatory Variables and adjust the numbering of the titles accordingly.

**Response:** Thank you very much for your careful review of our articles. After our inspection, we found that the title of the article was indeed repeated. We have changed 3.1.1 to "Explained variable", and the corresponding title of 3.1.2 is "Explained variable". We are very sorry for the oversight in our work.

Section the 2.1.3 needs to be edited more clearly.

**Response:** Thank you for your feedback. We have revised Section 2.1.3 (Path 3) to improve its clarity. We hope the changes address your concerns, and we appreciate your valuable input.

The authors ought to provide a clearer explanation for the justification behind their choice of (Debt ratio, economic cycle, marketability level, and industry capital intensity) as control variables.

**Response:** Thank you for your valuable advice. We have further explained the reasons for choosing these variables, provided details about the sources of variable data, and cited relevant literature in the revised manuscript. We hope this clarification meets your expectations, and we thank you for your insightful comments.

The study presented the outcomes of models 1 through 7 without a substantive discussion regarding the methodology employed to formulate these models.

**Response:** Thank you for your valuable feedback. We have restructured Section 3.2 (Model construction), and Section 4, (Analysis of results), providing explanations for the methods used in the models and discussing the results of each model. We hope this enhances the rigor of our academic research.

The study does not adequately emphasize the notable contributions it makes in comparison to prior research. Also, it lacks a section addressing its limitations and potential avenues for future research.

**Response:** Thank you for the valuable suggestions. With your assistance and guidance, the overall completeness of the paper has been enhanced. In the fifth section, we have added a discussion of this paper's contributions to the research field, while also addressing the limitations of the study and potential avenues for future research.

The level of language is adequate but it should be proofread before re-submission. Grammar should be revised before resubmitting the paper since several errors can be found in the text, especially with regard to prepositions, punctuation marks, and tenses. Also, avoid using personal pronouns in the academic paper.

**Response:** We appreciate your valuable feedback. In the revised manuscript, we have conducted multiple rounds of proofreading to address and rectify errors that were previously overlooked. This includes adding correct hyphenation, correcting preposition and punctuation errors, ensuring consistency in verb tenses, and avoiding the use of personal pronouns in academic papers, among other improvements.

In the question section, the authors filled in the following (The authors received no specific funding for this work) while at the end of the study, the authors wrote (Funding: The National Social Science Foundation Project of China, Grant/Award Numbers: 17XJY014). Please clarify this point.

**Response:** Thank you very much for your thorough review of our article. We have provided a revised explanation regarding the funding received in the resubmitted manuscript. We apologize for any oversights in the initial submission.

**Reviewer #2:** Positive Aspects:

- The article is well organized and provides valuable insights about research topic in the field. Moreover, the structure of the analysis is clear and logical.

- Research design is clear, comprehensive, and detailed. Also, the selection of variables is well justified and the integration between them and other factors enriches the analysis by explicitly examining the policy's effects on capacity utilization.

- Conclusions and implications provide a clear summary of the study's findings and offer actionable recommendations for policymakers.

**Response:** We appreciate your positive evaluation of our work. It is because of your valuable comments that the quality of this article had improved. Once again, I would like to express my sincere gratitude to you.

Suggestions for Improvements:

- Literature Review Section:

- Adding more examples can enhance the research. E.g., the researchers can incorporate some practical examples or case studies from China and/or other real-world examples to improve the research practicality and relevance. These of course should be recent examples and cases.

**Response:** Thank you for your valuable suggestions. Taking your valuable feedback into consideration, in the revised manuscript, we have included practical examples of overcapacity issues in other countries. Furthermore, we have conducted an in-depth analysis of cases in which other nations have implemented supply-side management to address overcapacity problems. We hope that these additions will enhance the practicality and relevance of our research.

- Research Design:

- Clarifying methodologies. For example, the use of a data envelope model is mentioned for measuring the capacity utilization rate. Therefore, providing more explanations and references regarding this model and how it's applied would enhance the clarity of the research design.

**Response:** Thank you for your valuable feedback. In response to your suggestions, we have provided further explanations of the data envelope model (DEA) in section 3.1.1 (Explained variables). We have also elucidated the reasons for selecting this model and provided a more detailed description of the process and results of using the DEA model to measure capacity utilization in the steel industry. We believe that these additions have improved the clarity of our research design.

- Expanding on Data Sources: For example, providing more information on the reliability, representativeness, and accuracy of the data to enhance the research design.

**Response:** Thank you for your constructive feedback. Based on your suggestions, in Section 3.1 (Variable selection), we have provided further elaboration on the rationale behind the selection of these variables and have offered additional information regarding the sources of each dataset. We have also included citations of relevant literature and viewpoints, with the aim of enhancing the reliability and representativeness of the data, thereby reinforcing the academic rigor and accuracy of this study.

- Discussing Limitations: For example, addressing potential limitations of the chosen methodologies, data sources, and the overall research design should add transparency and ensure a vigorous interpretation of results.

**Response:** Thank you for your valuable suggestions. In the third section (Research design), we have explained the methods employed, the rationale for their selection, and provided further elaboration on the sources of each dataset. In the fifth section (Conclusions), we have added a discussion about the contributions of this paper to the research field, while also addressing the limitations of the study and potential avenues for future research. We hope that these revisions will enhance the transparency of the article and ensure a robust interpretation of the results.

- Conclusions:

- Incorporating Quantitative Results: Incorporating specific quantitative results or statistics from the analyzed tests can enhance the credibility and clarity of the conclusions regarding the vitality of the chosen policy time breakpoint.

**Response:** Thank you for your constructive comments. We have incorporated specific quantitative results and statistics from the analyzed tests in the revised manuscript (Robustness test) to enhance the credibility and clarity of our conclusions regarding the chosen policy time breakpoint's significance. With your suggestions and help, the quality of the article has been greatly improved.

- Providing Citations for Theoretical Analysis: Providing references or citations to support the theoretical assertions can strengthen the argument and enhance academic and research rigor.

**Response:** Thank you for your valuable feedback. In response to your comments, we have reviewed the entire manuscript. We have provided further clarification on the constructed pathways and models, the propositions made, the choice of variables, data sources, and methodology. Additionally, we have included references to relevant literature and viewpoints. We hope these revisions enhance the rigor of the academic and research aspects.

- Elaborating more on Industry Cultivation Mechanism and multi-dimensional collaborative supervision: One of the research recommendations is that the need for improving the industry cultivation mechanism and multi-dimensional collaborative supervision. Expanding on what this entails and how it can positively impact capacity utilization would provide more clarity.

**Response:** Thank you for your valuable suggestions. We have re-edited the content in Section Five (Conclusions), and we hope that these revisions will enhance the completeness of the article.

- Limitations:

- Addressing Potential Limitations: Such as assumptions or constraints, to ensure a balanced view and promote transparency in the research.

**Response:** Thank you for your valuable feedback. Based on your suggestions, we have reorganized our paper and rewritten sections 2.1 (Analytical framework) and 4 (Analysis of results). In section 2.1 (Analytical framework), we have formulated six hypotheses concerning the impact and pathways of de-capacity policy on capacity utilization rate. In section 4 (Analysis of results), we have tested these six hypotheses through modeling. We hope that this revision will make the article more comprehensive and provide readers with a clearer perspective.

- Grammar and Style:

- There are minor grammatical errors. Stylistic improvements can be made to enhance the overall flow and readability of the text.

**Response:** Thank you for your thorough review and valuable feedback. In the resubmitted manuscript, we have conducted multiple checks for grammar and spelling throughout the entire document. We have also made corrections to errors that were previously overlooked, with the aim of enhancing the overall fluency and readability of the text.

**Reviewer #3:** In general, this is a well-written journal article. The authors have selected the "de-capacity" policy executed in China since 2010s, which is a topic that worth of discussing.

**Response:** We sincerely appreciate your positive feedback and are pleased that you found several strengths in our article. We believe that the "de-capacity" policy implemented in China since the 2010s is indeed a topic of significance and worthy of discussion.

However, some minor revisions should be made to extend the depth of this paper:

1. Please try to add some content on the Introduction and Literature review.

**Response:** We feel great thanks for your professional review work on our paper. In response to your feedback, we have made several improvements in the revised manuscript. Firstly, we have enhanced the content in the introduction section. Secondly, we have added practical examples of excess capacity issues in other countries, analyzed cases of supply-side management to address overcapacity, and included additional studies related to capacity utilization rate measurement in the references. Lastly, we have highlighted the shortcomings of existing literature and the contributions of our paper in addressing these research gaps. We believe that these enhancements further support and enrich our research.

2. In 2.2, please tell the readers the significance and the linkages of each path analysis.

**Response:** Thank you for your precious suggestions. To enhance reader comprehension, in Sections 2.1 (Analytical framework) and 2.2 (Path analysis), we conducted a more in-depth analysis of the five proposed paths, exploring their intrinsic connections, elucidating the basis for proposing these paths, and providing relevant references. We hope this will strengthen the scholarly aspect of the article.

3. Please re-check the fonts of the equations.

**Response:** Thank you for your thorough review. We apologize for our oversight. Based on your feedback, we have used the official PLOS ONE template provided on their website to ensure consistency in the formatting throughout the entire manuscript.

4. Please tell the research deficiencies & future works in the conclusion part.

**Response:** Thank you for your valuable suggestion. In response, we have addressed the research deficiencies and outlined potential areas for future work in the conclusion section. Your feedback has been valuable, and these improvements have been made accordingly.

5. Finally, please re-check the grammar, English expression and other details of the whole paper.

**Response:** Thank you for your precious suggestion. In the revised submission, we have conducted multiple checks for the grammar, English expression, and other details of the entire paper to ensure its quality. Your input is greatly appreciated.

**Reviewer #4:** The paper presents some interesting results, but it suffers from a lack of clarity and coherence. The objectives of the paper are not well defined and the flow of the paper is confusing. The paper would benefit from a thorough revision of the structure and the language. There are also many typographical errors that need to be corrected.

**Response:** Thank you for recognizing the work done in our article. Based on your feedback, we have made the following revisions:

- Firstly, we have restructured the paper. In the section 1 (Introduction), we have clarified the research objectives, and in the second section 2.1 (Analytical framework), we have explained the analytical framework. Throughout the subsequent sections, we have made various additions, deletions, and modifications to enhance the paper's clarity and coherence.
- Secondly, we conducted multiple reviews to rectify grammar and spelling errors, including tenses, punctuation, and prepositions, among others.
- Lastly, in the resubmitted manuscript, we used the official PLOS ONE style templates to ensure consistent formatting throughout the paper.

Once again, we sincerely appreciate your valuable feedback, which has significantly contributed to the improvement of the quality of our manuscript.

**Reviewer #5:** abstract needs an introductory statement. the author starts with data data duration and discussing the model.

methodology is not clear in abstract

results of the work should be clear in the abstract as well

**Response:** Thank you for your valuable feedback. We appreciate your input, and we have made the following revisions to the abstract to address your concerns:

- In the revised abstract, we have included an introductory statement to provide context for the reader. We now begin by briefly introducing the research topic before discussing the data and the duration of the study.
- We have also clarified the methodology used in the abstract, giving a concise overview of the approach taken in the study.
- Furthermore, we have made sure to include a clear statement about the results of the work in the abstract, summarizing the key findings to give readers a better understanding of the paper's contributions.

Thank you again for your valuable advice. We hope these improvements enhance the clarity and comprehensibility of the abstract.

literature Review discusses the issue of over capacity and suggested solutions but only for China. it is clear that the data is for China but there should be literature for other countries and what other countries suggested as solutions to this problems' which the authors can reflect on in their proposed solutions.

**Response:** Thank you for your thorough review of our paper. In the revised manuscript, we have enriched the content of the references by adding real-world examples of excess capacity issues in other countries. We have also conducted an analysis of cases where other countries implemented supply-side management to address excess capacity problems. These cases have been instrumental in shaping our arguments and viewpoints presented in this paper. We hope that these enhancements contribute to the overall quality of the article.

equations 1 to 3 parameters' are not clear the letters are tangled please fix this formation issue please, what is w* ** , alpha, beta and omega what do you mean by elasticity coefficients?

for the same suggested model equations 1 to 3. the paragraph after it describes the model parameters in the past tense. it should be in present tense

the whole model explanation is not clear and needs to be rewritten in clear way

**Response:** Thank you for your question, and I apologize for the issues in our work. In response to your inquiry, in the revised manuscript (Model Construction), we have thoroughly reorganized the model, carefully analyzed the meaning of each variable, and explained the source of each model. Furthermore, we have reviewed the language in this section and corrected any tense-related errors. We hope that these efforts will enhance the rigor of our academic and research work.

section 3.3: refer to those references in the table, yet again those steel industry year books are they public information and can they be listed in the references? even if data is interpolated you should indicate that in the table data

**Response:** Thank you for your questions and valuable suggestions. In sections 3.1 (Variable selection), we have provided further explanations regarding the sources of each piece of data, supported by relevant literature, which include industry reports and documentary data. Furthermore, in section 3.3 (Data sources), we have summarized the data sources more comprehensively. We believe that these efforts enhance the comprehensiveness of the paper.

sec. 4.1 you start by explaining table 3 but how did the 7 models were obtained? and why are they considered models it is not clear? which equations 1 to 3 are used, give example to that for the data in table 3.

**Response:** Thank you for your feedback. Regarding your comments: In Section 4 (Analysis of results), the models we obtained are all based on Equations 5 to 7. In the resubmitted manuscript, in Section 4 (Analysis of results), we have provided further explanations on how these models were derived. Additionally, we have rewritten Section 4 and provided examples of the data in the table. We hope that this work will present the results of the paper in a clearer manner. We hope that these clarifications address your concerns.

after verifying the models listed, the data analysis is also better explained in plots. the section is long and with large paragraphs and needs major rewriting.

**Response:** Thank you for your feedback. We agree that the section is lengthy with extensive paragraphs and requires significant rewriting. We have rewritten the section 4 (Analysis of results), explaining the sources of each model and discussing the results of each model. We hope this improves the readability of the paper.

sec. 4.2: start by explaining the fist method placebo test, was this listed in the methods? and how was it used for years other than 2015 as other years are mentioned. later in the same long paragraph it is mentioned that 2014 and 2016 only were used this requires to explain why only those years and it is worth it to see the effect of the proceeding years.

**Response:** Thank you for your questions and valuable suggestions. We have incorporated specific quantitative results and statistics from the analyzed tests in the revised manuscript (Robustness test) to enhance the credibility and clarity of our conclusions regarding the chosen policy time breakpoint's significance. With your suggestions and help, the quality of the article has been greatly improved.

conclusion is too long and in one paragraph

**Response:** Thank you for your valuable feedback. We have reorganized the conclusion section as per your suggestions by making it more concise. This adjustment has helped to better present our research findings and viewpoints while ensuring the conclusion is more structured and succinct.

Thanks again for your comments and suggestions. I apologize for the inadequacy of my work, if you have any questions please contact me.

Sincerely,

Hao Li

[202121000581@stu.swpu.edu.cn](mailto:202121000581@stu.swpu.edu.cn)
